# The Simple Act of Waiting: Natural Language Processing in the Identification of In‐Hospital Delays

**DOI:** 10.1111/hex.70017

**Published:** 2024-09-30

**Authors:** Daksh Tyagi, Sheryn Tan, Charis Tang, Joshua Kovoor, Aashray Gupta, WengOnn Chan, Samuel Gluck, Toby Gilbert, Andrew C. Zannettino, Patrick G. O'Callaghan, Stephen Bacchi

**Affiliations:** ^1^ School of Medicine and Public Health University of Newcastle Callaghan New South Wales Australia; ^2^ Faculty of Health and Medical Sciences University of Adelaide Adelaide South Australia Australia; ^3^ Ballarat Base Hospital Ballarat Central Victoria Australia; ^4^ Royal North Shore Hospital St. Leonards New South Wales Australia; ^5^ Royal Adelaide Hospital Adelaide South Australia Australia; ^6^ Lyell McEwin Hospital Elizabeth Vale South Australia Australia; ^7^ College of Medicine and Public Health Flinders University Bedford Park South Australia Australia

We read with interest the study by Abdelhalim et al. (DOI:10.1111/hex.14050) that addressed the persistent challenge of delayed hospital discharge (DHD) by identifying its primary causes and emphasising the significance of effective communication and management in healthcare settings [1]. This has the effect of prolonging hospital stays, increasing costs, reducing capacity and poorer patient outcomes [2, 3, 4, 5].

One of the main findings of the study is that poor communication—between hospitals, community health and social care providers, and within hospital departments—was consistently a factor in DHD [1]. Numerous articles highlighted the inadequate communication and coordination among hospital staff, patients and healthcare and community institutions acting as a significant obstacle to minimising DHD. Additionally, many research findings agreed that the primary cause of the DHD issue lies in hospital management, internal processes and inadequate coordination [1].

In line with this, we conducted a multicentre retrospective cohort study evaluating consecutive general medicine inpatient admissions to two metropolitan tertiary hospitals over a 2.5‐year period using natural language processing (NLP) to evaluate documented discharge delays.

Our findings provide a South Australian inpatient general medicine complement to the 700 studies spanning the last 24 years analysed by Abdelhalim et al. Evaluation of 28,377 unique visits and associated 193,251 individual ward round notes suggested significant association between the use of a delay phrase (‘wait’ and ‘chase’) in a ward round note and greater length of stay (LOS). Among the individual hospital processes evaluated, the term ‘wait’ was frequently linked with magnetic resonance imaging (MRI) and computed tomography (CT), whereas the term ‘chase’ was frequently linked with blood tests.

Our study demonstrated that it is feasible to use NLP on medical free text to gain insights into reasons associated with DHD. Patients who had ward round notes with specific delay phrases had longer LOS. This method can also be used to identify hospital processes that are frequently discussed in the context of these described delays. This type of analysis may present novel insights into aspects of the psychology of medical officers. Results that typically have a shorter turnaround time, such as blood and urine tests, were discussed more in the context of ‘chasing’, whereas other test results, such as MRI and CT, were more often discussed in the context of ‘waiting’.

Although the results of the study are novel, their utility has not yet been investigated. Some identified delays may not be amenable to change. However, there may be delays amenable to change in specific circumstances. For example, when an access‐block crisis point is reached, a hospital system may respond by leveraging additional resources to overcome this situation. An automated and rapid evaluation of already described factors contributing to hospital delays for current inpatients may be able to direct such resources, such as extended access to radiological services.

Identification of the reasons behind DHD is absolutely crucial in streamlining the outflow, and by extension, the overall load of patients upon a hospital. One of the primary rationales behind conducting the scoping review by Abdelhalim et al. was to illustrate that rather than solely focusing efforts on capacity expansion, we should advocate for hospital management enhancements and interventions targeting foundational issues, such as social and transitional care support [1].

The study conducted by our research team further shows that there is a wide scope for research in this field. Further research may seek to examine the generalisability of these findings and methods to other specialties and centres. Studies examining the potential utility of this information should be considered. Such studies may seek to conduct a cross‐sectional analysis at a time of increased healthcare demand to determine whether these methods may identify delays amenable to modification.

Similar to the findings of Abdelhalim et al., our results also highlight the need for increased research focus on the reasons behind DHD, as opposed to the current direction of funding, which is more focused on capacity expansion [1].

## Author Contributions

**Daksh Tyagi:** conceptualisation, data curation, formal analysis, investigation, methodology, project administration, writing of the draft, writing–review and editing. **Sheryn Tan:** conceptualisation, formal analysis, investigation, methodology, project administration, supervision, validation, writing–review and editing. **Charis Tang:** conceptualisation, formal analysis, investigation, methodology, project administration, supervision, validation, writing–review and editing. **Joshua Kovoor:** conceptualisation, formal analysis, investigation, methodology, project administration, supervision, validation, writing–review and editing. **Aashray Gupta:** conceptualisation, formal analysis, investigation, methodology, project administration, supervision, validation, writing–review and editing. **WengOnn Chan:** formal analysis, investigation, methodology, project administration, supervision, validation, writing–review and editing. **Samuel Gluck:** conceptualisation, formal analysis, investigation, methodology, project administration, supervision, validation, writing–review and editing. **Toby Gilbert:** formal analysis, investigation, methodology, project administration, supervision, validation, writing–review and editing. **Andrew C. Zannettino:** formal analysis, investigation, methodology, project administration, supervision, validation, writing–review and editing. **Patrick G. O'Callaghan:** conceptualisation, formal analysis, investigation, methodology, project administration, supervision, validation, writing–review and editing. **Stephen Bacchi:** conceptualisation, formal analysis, investigation, methodology, project administration, supervision, validation, writing–review and editing.

## Conflict of Interest

The authors declare conflicts of interest.

## Data Availability

Data can be made available via request to the authors.
